# A Spectral Confocal Measurement Method for High-Aspect-Ratio Deep Holes Based on Stepped Ring Gauge and Hierarchical Error Compensation

**DOI:** 10.3390/s26113384

**Published:** 2026-05-27

**Authors:** Yao Liu, Gui Wang, Daguo Yu, Huifu Du

**Affiliations:** 1 School of Mechanical Engineering, North University of China, Taiyuan 030051, China; bz20240202@st.nuc.edu.cn (Y.L.); yudaguo@nuc.edu.cn (D.Y.); b20230211@st.nuc.edu.cn (H.D.); 2 Shanxi Key Laboratory of Smart Manufacturing and Intelligent Testing for Pipeline and Hole Structures in Harsh Environments, Taiyuan 030051, China; 3 Shanxi North Maching-Building Co., Ltd., Taiyuan 030051, China

**Keywords:** spectral confocal measurement, deep-hole inspection, hierarchical error compensation, system calibration, stepped ring gauge

## Abstract

To address the issues of uneven accuracy across the entire hole depth and profile distortion caused by multi-source errors in spectral confocal deep-hole measurement, this paper proposes a measurement method involving global calibration using a stepped ring gauge and hierarchical compensation for multi-source errors. By classifying core measurement errors into three categories—geometric deviation, structural error, and dynamic process error—according to their propagation laws, this paper establishes a progressive comprehensive compensation system comprising “geometric calibration–structural correction–dynamic filtering”. Specifically, using a stepped ring gauge as the reference, the system’s intrinsic geometric parameters are identified via the Levenberg–Marquardt (LM) algorithm; structural errors introduced by the deflection of components due to self-weight are quantitatively corrected based on a statics model; periodic harmonic errors are sequentially separated; random noise is effectively suppressed by combining least-squares harmonic fitting with adaptive wavelet threshold filtering. Experimental results demonstrate that this method can limit the maximum absolute deviation in the inner diameter measurement of standard ring gauges to within 0.2 μm, stabilizing the measurement repeatability over the entire depth of deep-hole workpieces with length-to-diameter ratios exceeding 30:1 to within 0.8–1.6 μm, with an expanded uncertainty of U = 3.8 μm (k = 2). This method enables the precise reconstruction of deep-hole inner wall topography, providing a highly versatile technical foundation and implementation scheme for the high-precision non-destructive testing of deep holes with large length-to-diameter ratios.

## 1. Introduction

In advanced manufacturing sectors such as aerospace, precision hydraulics, and high-end molding, deep-hole components—including aircraft engine fuel nozzles, hydraulic valve bodies, and precision mold cooling channels—serve as core elements to perform critical functions. The geometric and dimensional accuracy, as well as the surface topography of their inner walls, directly determines the products’ in-service performance, operational reliability, and service life [1,2]. Such components are typically characterized by a small bore diameter and a large length-to-diameter (L/D) ratio. The narrow internal cavities form an enclosed measurement space that is visually inaccessible and difficult for probes to access. High-precision full-circumferential inspection of the inner walls over the entire hole depth has long been a well-recognized technical challenge in the industry [3].

Traditional deep-hole inspection relies primarily on contact measurement methods. Although equipment such as internal micrometers, coordinate measuring machines (CMMs), and inductive micrometers can perform basic dimensional inspection, this type of equipment presents significant limitations in engineering applications: contact probes are prone to scratching precision inner walls, making it difficult to obtain complete cross-sectional topography data [4]; under conditions of large L/D ratios, the self-weight deflection of the measuring rod causes measurement errors to increase sharply with hole depth [5]; single-point contact modes struggle to achieve efficient full-circumferential scanning of the topography, and the contact force can easily induce additional deformation on the measured surface, making it difficult to effectively control dynamic measurement errors [6]. To overcome these bottlenecks, non-contact measurement technology has become a key research focus in deep-hole inspection [7]. However, existing mainstream non-contact solutions all exhibit limitations in their adaptability to deep-hole scenarios: although laser triangulation offers fast response, it is susceptible to tens of micrometers of nonlinear distortion in deep holes due to optical occlusion and internal reflections [8,9]; in small-diameter deep holes (e.g., D < 10 mm, L/D > 30), machine vision is restricted by a short depth of field and illumination attenuation, typically degrading spatial resolution beyond 50 μm [10]; constrained by beam divergence, ultrasonic inspection generally yields a sub-millimeter spatial resolution (0.8 mm), failing to resolve micron-level topographical features [11].

In recent years, spectral confocal displacement sensing technology has emerged as an ideal solution for high-precision inner wall inspection of small-diameter, large L/D ratios deep holes, owing to its advantages of non-contact measurement, high axial resolution, insensitivity to measured surface tilt, and strong resistance to ambient light interference [12,13]. Existing research has largely focused on the development of miniaturized probes, the integration of motion systems, the optimization of optical performance, and the development of inner wall topography reconstructing algorithms [14,15,16,17]. Despite these research advances, these approaches generally suffer from high costs, complex system architecture, and poor on-site adaptability. The fundamental cause lies in the lack of systematic research into the coupling mechanisms and propagation laws of multi-source errors during deep-hole measurement [18]. Specifically, existing research has neither conducted systematic modeling and decoupling analysis of multi-source errors—such as assembly eccentricity, self-weight deflection of components, and dynamic motion disturbances—nor established a comprehensive calibration and error control system covering the entire “geometric–structural–dynamic” process [19]. Consequently, while the measurement system can maintain theoretical accuracy in the shallow hole section, errors accumulate continuously as the probe enters the middle and deep sections of the hole, leading to a drastic degradation in measurement accuracy. This ultimately causes the aforementioned issues of uneven accuracy and topographical distortion, rendering it impossible to translate the theoretical measurement accuracy of the spectral confocal sensor into actual measurement performance across the entire hole depth.

To this end, this paper proposes a measurement method based on global calibration using a stepped ring gauge and hierarchical compensation for multi-source errors, aimed at addressing the engineering challenge of translating the theoretical accuracy of spectral confocal sensors into actual measurement accuracy across the entire bore depth. Without modifying the existing hardware, the following three progressive research tasks are undertaken: firstly, constructing a parametric model of multi-source errors; secondly, proposing a global calibration method based on a stepped ring gauge; and finally, establishing a progressive, hierarchical comprehensive compensation system. Through calibration experiments and verification via actual workpiece measurements, the proposed method effectively mitigated the issue of uneven accuracy across the full bore depth, ultimately providing a low-cost, highly versatile technical solution for the high-precision non-destructive testing of deep holes with high aspect ratios.

## 2. Measurement System and Error Source Analysis

### 2.1. Measurement System

**Figure 1:**
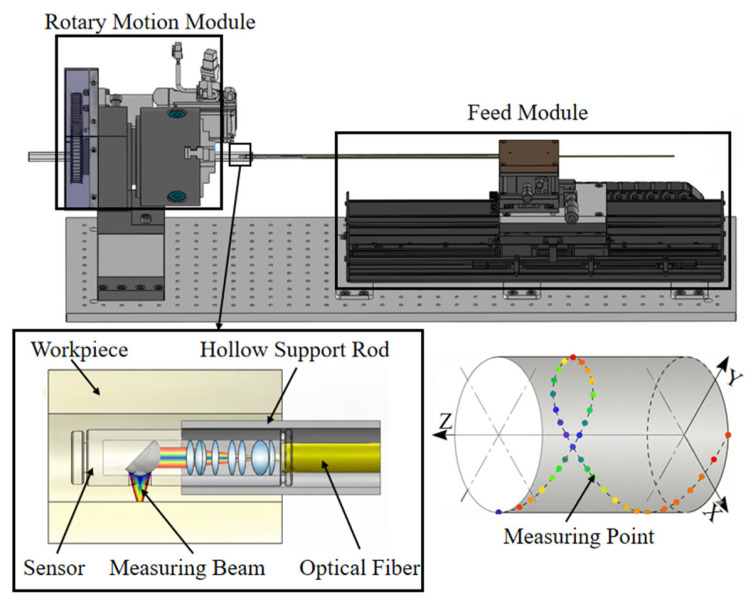
Schematic of the spectral confocal measurement device and unified coordinate system for inner wall of high-aspect-ratio deep hole.

To address the measurement challenges associated with the inner walls of small-diameter deep holes, this paper presents the design of a precision measurement device based on the spectral confocal principle, as shown in Figure 1. The device is mainly composed of two core motion modules: a linear feed module and a rotary motion module. The linear feed module adopts a maglev linear motor to directly drive the spectral confocal sensor along the deep hole axis, thereby enabling precise axial positioning of the sensor. The rotary motion module is driven by a high-precision servo motor, and motion is transmitted to the workpiece clamping end through a two-stage transmission chain comprising a planetary gearbox and a synchronous pulley. This transmission system drives the deep-hole workpiece to rotate at a constant speed, enabling full-circumferential 360° scanning of the deep-hole inner wall. Throughout the measurement process, the sensor and the workpiece maintain a non-contact state, effectively avoiding additional deformation and frictional interference caused by contact measurement methods, thereby significantly improving the dynamic response and measurement stability of the system.

The device integrates a spectral confocal displacement sensor inside a hollow support rod, with the sensor driven by a linear motor, with its measurement beam emitted perpendicular to the support rod axis. Using the spectral confocal principle—which exhibits a one-to-one correspondence between wavelength and axial focal position—the system extracts the peak wavelength of the light reflected from the deep hole inner wall, thereby achieving high-precision non-contact measurement of the inner wall topography.

To clearly define the spatial measurement coordinate relationships, a unified O-*XYZ* Cartesian coordinate system is established: the origin is located at the center of the end face at the workpiece clamping end; the rotation axis of the workpiece is defined as the *Z*-axis (corresponding to the sensor feed direction into the hole); the *X*-axis denotes the horizontal radial direction; the *Y*-axis denotes the vertical radial direction (opposite to the gravitational direction); the XOY plane forms the radial measurement cross-section perpendicular to the aforementioned hole axis. All length units in this coordinate system are specified in millimeters (mm).

### 2.2. Error Source Analysis

According to their generation mechanisms, spatiotemporal distribution characteristics, and their propagation laws along the full depth of the hole, the core measurement errors are classified into three categories: geometric deviation induced by assembly and positioning deviations, structural error caused by the inherent structural characteristics of the measurement system, and dynamic process error caused by dynamic time-varying disturbances during measurement motion.

#### 2.2.1. Geometric Deviations

**Figure 2:**
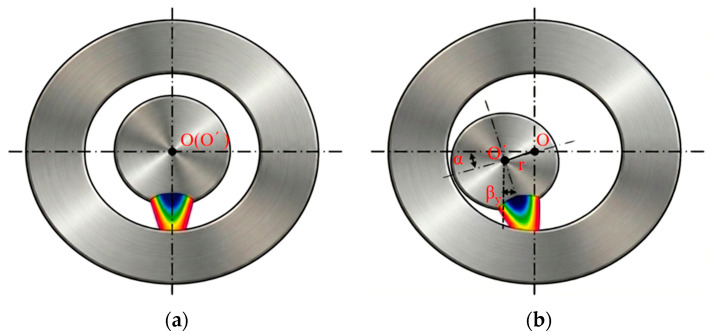
Comparison between ideal deviation-free measurement state and actual measurement state with geometric eccentricity and pose deflection in radial section of deep hole: (**a**) Ideal deviation-free measurement state; (**b**) Actual measurement state with geometric eccentricity and pose deflection.

The ideal radial measurement cross-section is shown in Figure 2a: the feed axis of the spectral confocal displacement sensor is perfectly coaxial with the theoretical axis of the deep-hole workpiece, and the displacement value measured by the sensor can be directly used to reconstruct the deep-hole inner wall topography. However, limited by the sensor’s measurement range and system assembly accuracy, the actual radial measurement cross-section is shown in Figure 2b: an angular deviation $\beta_{y}$ exists between the sensor’s measurement optical axis and the hollow support rod axis; meanwhile, the sensor feed axis and the workpiece axis cannot achieve perfect coaxial alignment. These deviations generate a radial eccentricity r and an eccentricity angle α, which ultimately introduces significant geometric eccentricity errors.

#### 2.2.2. Structural Errors

**Figure 3:**
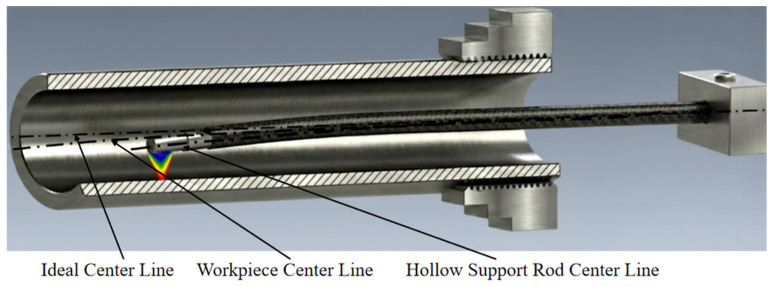
Schematic of the mechanism of structural error caused by self-weight deflection of hollow support rod and measured workpiece.

Structural errors primarily arise from elastic deformation caused by the self-weight of key components, and the resulting changes in the spatial measurement relationships are shown in Figure 3. First, the hollow support rod holding the sensor undergoes deflection under its own weight, causing the spatial pose of the measurement beam emitted by the sensor to deviate from the theoretical axis of the workpiece and directly leading to distortion of the measured distance. Second, the clamped deep-hole workpiece also undergoes deflection due to self-weight, causing the theoretical center of the measured cross-section to undergo continuous radial shift along the hole depth, with the magnitude of this workpiece deflection increasing nonlinearly with hole depth. These two forms of deflection collectively alter the predefined theoretical spatial geometric relationship between the measurement beam and the deep-hole inner wall, thereby introducing non-negligible systematic structural errors.

#### 2.2.3. Dynamic Process Errors

Dynamic process errors arise from time-varying motion disturbances of the measurement system, which mainly manifest as high-frequency random noise and periodic harmonic interference. Their generation mechanisms fall into two main categories. First, the inevitable vibrations generated during the feed motion of the maglev linear motor are directly transmitted to the sensor, causing high-frequency small-amplitude shifts in the focal point of the measurement beam, thereby introducing components unrelated to the true inner wall topography into the measured distance signal. Second, non-ideal characteristics of the rotary transmission chain—such as backlash, time-varying stiffness, and motion instability—cause the motion errors of the servo motor to be periodically transmitted to the rotational motion of the workpiece through the two-stage reduction and belt transmission, generating rotational speed-dependent harmonic components in the measurement signal. These two types of dynamic disturbances are coupled with each other in the measurement signal, ultimately introducing interference unrelated to the true inner wall topography. Of these two types of interference, the harmonic interference is strictly synchronized with the workpiece rotational frequency and is a low-frequency deterministic disturbance, while the random noise is a high-frequency broadband disturbance, and the two can be completely separated in the frequency domain.

These three types of errors transmit and superimpose sequentially through the measurement process, exhibiting distinctly different evolutionary characteristics. Consequently, their quantitative contribution to the overall error varies dynamically with the hole depth. Specifically, geometric deviation constitutes a fixed systematic error and accounts for the predominant proportion of the error in the shallow region of the hole. Structural error, however, accumulates and amplifies non-linearly with increasing depth; at extreme depths, its absolute magnitude exceeds that of geometric deviation, making it the most significant source of error. Meanwhile, the dynamic process error manifests as a random disturbance across the entire depth range. Driven by the dynamic shifts in relative significance and the distinct characteristics of these three types of errors, this paper employs a hierarchical and progressive compensation strategy to achieve consistent accuracy across the entire hole depth.

## 3. System Calibration and Error Compensation

To systematically address the aforementioned three types of measurement errors, this paper proposes the core principle of “model-driven, software-based compensation” to establish a progressive, comprehensive compensation system comprising “geometric calibration–structural correction–dynamic filtering”. Specifically, for fixed systematic geometric deviations, the precise calibration of the intrinsic geometric parameters of the measurement system is performed using a stepped ring gauge; for structural errors that accumulate nonlinearly with increasing hole depth, quantitative correction is achieved using a static self-weight deflection model; and for dynamic disturbances over the entire hole depth, noise suppression and signal refinement are implemented using a hierarchical frequency-domain signal processing method.

### 3.1. Calibration and Calculation of Geometric Deviation Parameters

**Figure 4:**
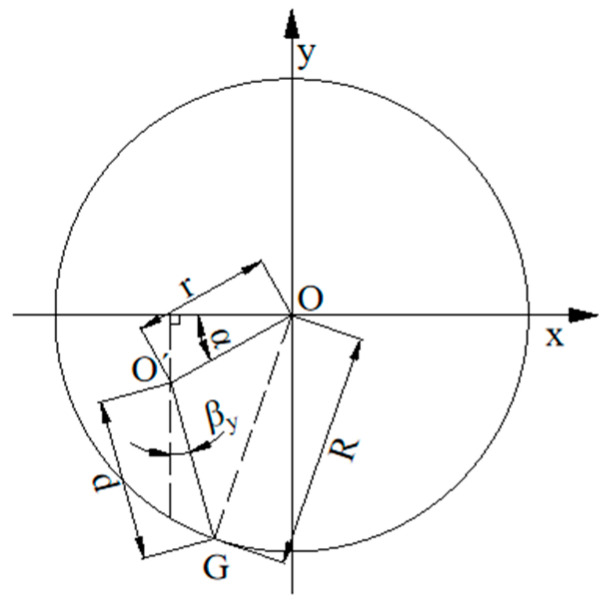
Parametric analytical model of geometric deviation in deep hole measurement with radial eccentricity and pose deflection.

The geometric deviation represents a fixed systematic error in spectral confocal deep-hole measurement, whose precise compensation depends on the robust identification of these key parameters, including system eccentricity and sensor spatial pose. To achieve this precise compensation, this paper first establishes a parametric analytical error model of geometric deviation, as shown in Figure 4.

In the model, the sensor’s measurement center O’ exhibits a radial eccentricity r and an eccentricity angle α with respect to the theoretical rotational center O of the workpiece. The measurement beam emitted by the sensor has a radial angular deviation $\beta_{y}$ and is focused at point G on the deep-hole inner wall, yielding a measurement distance d. The true radius of the workpiece at point G is R. Using the law of cosines for △OGO’, we derive the analytical expression of the geometric deviation error as follows:

(1) $$R=\sqrt{r^{2}+d^{2}-2drsin(\beta_{y}-\alpha )}$$

**Figure 5:**
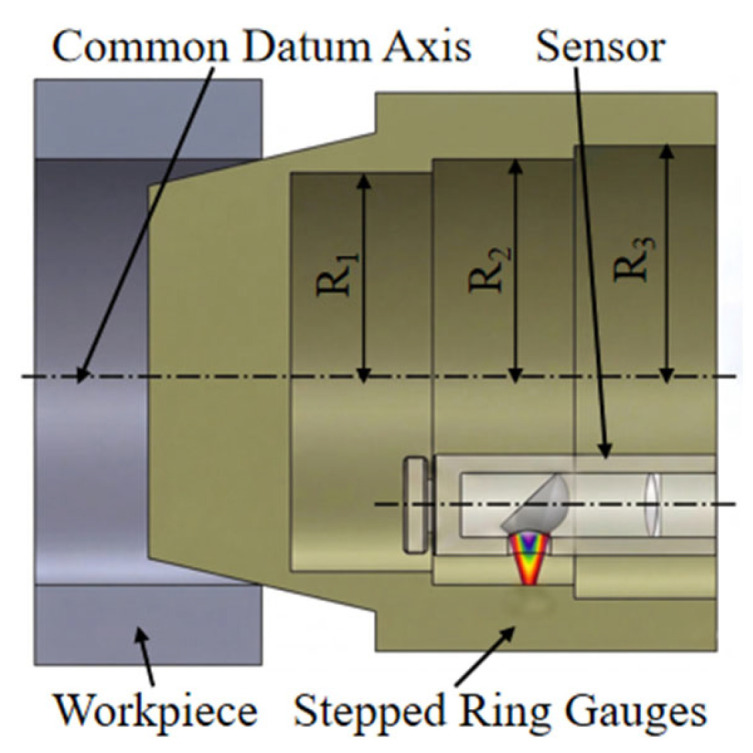
Schematic of the multi-step coaxial ring gauge for global system calibration.

Traditional single-ring gauge calibration schemes can only provide α single reference radius R and are unable to solve an underdetermined system of equations comprising multiple unknown parameters, resulting in inherent limitations including non-unique calibration solutions and weak anti-interference robustness. To address this issue, this paper presents the design of a coaxial multi-step stepped ring gauge to serve as the calibration reference, as shown in Figure 5. The inner cylindrical surface of each step has a pre-calibrated high-precision radius ${\;R}_{i}$, which provides multiple independent sets of calibration reference values ($d_{i},{\;R}_{i}$). This multi-reference calibration strategy transforms the original underdetermined identification problem into a nonlinear overdetermined system of equations with a unique optimal solution.

Further analysis reveals that the eccentricity angle α and angular deviation $\beta_{y}$ appear in the radius calculation only in their combined form $\beta =\beta_{y}-\alpha$, eliminating the need to identify the two parameters separately. Based on this conclusion, this paper designates the combined angle β and the radial eccentricity r as the core parameters to be calibrated, and constructs a nonlinear least-squares (NLS) optimization model:

(2) $$\min F(r,\beta )=\frac{1}{2}{\sum_{i=1}^{n}\left[R_{i}-\sqrt{r^{2}+d_{i}^{2}-2d_{i}r\sin(\beta )}\right]}^{2}$$

To solve this nonlinear optimization model globally, this paper employs the Levenberg–Marquardt (LM) algorithm, first proposed in [20]. With an adaptive damping factor introduced, this algorithm balances the rapid convergence of the Gauss-Newton method and the global convergence capability of the steepest gradient descent method, thereby effectively preventing iterative divergence, and exhibits robust numerical stability. The core iterative expression of the algorithm is given as follows:

(3) $$(J^{T}J+\mu I)\delta =-J^{T}f$$

where J denotes the Jacobian matrix of the nonlinear residual function of the optimization model, μ denotes the adaptive damping factor, I denotes the identity matrix of corresponding dimensions, δ denotes the increment vector of the parameters to be identified, and f denotes the corresponding nonlinear residual vector.

Through iterative optimization using the LM algorithm until convergence, the globally optimal identification results for the radial eccentricity r and the combined angle β are obtained. As fixed system constants, these geometric deviation parameters can be applied independently once identified. Furthermore, the numerical properties of the LM algorithm ensure that the identification process exhibits high stability and robustness against variations in initial estimates.

### 3.2. Modeling and Correction of Structural Errors

Structural error is the dominant cumulative systematic error responsible for the sharp decline in measurement accuracy with increasing hole depth, especially in the middle and deep sections of the hole. It essentially arises from the elastic deformation of the support rod and the workpiece under their own weight, and this deformation breaks the predefined theoretical spatial geometric relationship between the measurement beam and the deep-hole inner wall, ultimately leading to measurement distance distortion. Most existing studies have only considered the self-weight deflection of a single component and have failed to establish a coupled correction model for the self-weight deflections of both the support rod and the workpiece. Consequently, it is still impossible to achieve full-range precise compensation for structural error over the entire hole depth.

Based on the Euler–Bernoulli beam theory [21], this paper establishes separate models for the self-weight bending deflection of the hollow support rod and the deep-hole workpiece and ultimately constructs a coupled error correction model for the two components. The basic modeling assumptions are as follows: both the support rod and the workpiece are homogeneous elastic bodies with constant cross-sections, satisfying the small-deformation assumption of the Euler–Bernoulli beam theory; the clamped end of each component is subject to a fully fixed constraint, with no looseness or additional deformation; gravity is the only static load considered in this model, and the effects of assembly stress and thermal deformation are ignored. Benefiting from the constant temperature (20 ± 1 °C), the ultra-low expansion carbon fiber rod, and workpiece annealing, order-of-magnitude assessment shows that thermal and stress deformations (0.044 μm) are far below the linear motor’s repeatability (1 μm). Therefore, they are neglected as higher-order small quantities in the macroscopic modeling.

By modeling the hollow support rod as a cantilever beam with a concentrated tip load from the spectral confocal sensor at the beam’s free end, this paper derives an analytical calculation model for the free-end deflection angle of the rod under the coupling effect of the support rod’s self-weight and the concentrated end load:

(4) $$\theta_{s}=\frac{32m_{h}g{L_{s}}^{2}}{\pi E_{s}({D_{s}}^{4}-{d_{s}}^{4})}+\frac{8g\rho_{s}{L_{s}}^{3}}{3E_{s}({D_{s}}^{2}+{d_{s}}^{2})}$$

where $m_{h}$ denotes the mass of the spectral confocal sensor at the rod’s free end (kg); $L_{s}$ denotes the cantilever length of the support rod (m); $D_{s}$ and $d_{s}$ denote the outer and inner diameters of the support rod (m), respectively; $\rho_{s}$ denotes the density of the support rod ($kg/m^{3}$); $E_{s}$ denotes the elastic modulus of the support rod (Pa); and g denotes the gravitational acceleration ($m/s^{2}$).

By modeling the deep-hole workpiece as a uniform cantilever beam with a fully fixed clamped end and defining the sensor’s axial feed depth z as the independent variable along the workpiece rotational axis, this paper establishes the theoretical workpiece’s self-weight deflection distribution function over the full axial length of the hole:

(5) $$\delta_{w}\left(z\right)=\{\begin{array}{ll} 0 & 0\leq z<a\\ \frac{2\rho_{w}g{\left(z-a\right)}^{2}}{3E_{w}({D_{w}}^{2}+{d_{w}}^{2})}\left[{\left(z-a\right)}^{2}-4(L_{w}-a)\left(z-a\right)+6(L_{w}-a{)}^{2}\right] & a\leq z\leq L_{w} \end{array}$$

where $L_{w}$ denotes the total effective length of the workpiece (m); $D_{w}$ and $d_{w}$ denote the outer and inner diameters of the workpiece (m), respectively; $\rho_{w}$ denotes the density of the workpiece ($kg/m^{3}$); $E_{w}$ denotes the elastic modulus of the workpiece (Pa); a denotes the fixed clamping length of the workpiece at the support end (m); and z denotes the axial feed position of the sensor along the hole depth (m).

By incorporating the coupling error mechanism between the deflection angle $\theta_{s}$ of the support rod (which causes deflection of the measurement beam) and the deflection $\delta_{w}\left(z\right)$ of the workpiece (which leads to a shift in the measurement datum), this paper derives the correction formula at the rotation angle θ, which relates the raw radial measurement value $d_{raw}(\theta )$ to the structural error compensated value $d_{struct}(\theta )$ as follows:

(6) $$d_{struct}(\theta )=d_{raw}(\theta )\cdot \cos\theta_{s}-\delta_{w}\left(z\right)$$

### 3.3. Suppression and Filtering of Dynamic Process Errors

Dynamic process error is time-varying, coupled random disturbances distributed over the entire hole depth, which mainly manifest as high-frequency random noise induced by feed vibrations and periodic harmonic interference introduced by the rotary drive train, which is synchronized with the rotation angle of the workpiece. Most existing studies only adopt single-stage filtering methods to address dynamic process error, without incorporating mechanism-based error separation. This single-stage filtering strategy tends to cause the loss of real topographical feature details of the deep-hole inner wall and struggles to balance the precise removal of periodic harmonic components and the effective suppression of random noise. Accordingly, this paper proposes a strategy of “mechanism-guided analysis, progressive signal processing” to sequentially quantify the feed vibration characteristics, globally identify the rotational harmonic interference components, and implement detail-preserving random noise suppression, thereby achieving full-range precise compensation for dynamic process error.

#### 3.3.1. Mechanism Analysis and Frequency-Domain Characteristic Quantification of Feed Vibration

The primary excitation source of feed vibration is the normal-direction (perpendicular to the feed direction) force fluctuation of the maglev linear motor. This feed vibration is transmitted to the sensor through the mounting base, inducing micro-displacements of the focal point of the measurement beam. This paper establishes a dynamic model of the feed system by treating it as a single-degree-of-freedom (SDOF) forced vibration system. We perform data acquisition during the constant-velocity feed phase, specifically after the transient vibration components have fully decayed and the system has entered the steady-state response phase. Based on linear forced vibration theory, this paper derives the amplitude of the steady-state displacement response under harmonic excitation as follows [22]:

(7) $$Y=\frac{F_{0}/k}{\sqrt{{\left[1-(f_{z}/f_{n}{)}^{2}\right]}^{2}+{\left[2\zeta f_{z}/f_{n}\right]}^{2}}}$$

where $F_{0}$ denotes the amplitude of the normal-direction force fluctuation of the maglev linear motor; k denotes the equivalent stiffness of the SDOF feed system; $f_{z}$ and $f_{n}$ denote the excitation frequency of the maglev linear motor’s normal force fluctuation and the natural frequency of the feed system, respectively; and ζ denotes the equivalent damping ratio of the feed system.

Both theoretical analysis and experimental verification demonstrate that, through structural optimization to increase the natural frequency of the system, the condition $f_{z}/f_{n}\ll 1$ is satisfied within the full rated operating range of the system. Consequently, the system consistently operates within the stiffness-controlled region, the dynamic amplification factor approaches unity, and the steady-state vibration amplitude is strictly controlled to within the sub-micrometer level. This steady-state vibration manifests as high-frequency broadband random noise that is independent of the real inner-wall topography and can be effectively isolated via subsequent wavelet threshold filtering [23].

#### 3.3.2. Global Identification and Elimination of Rotational Harmonic Errors Based on Least-Squares Harmonic Fitting

The non-ideal transmission characteristics of the rotary drive train—such as transmission backlash, time-varying meshing stiffness, and rotational motion non-uniformity—introduce periodic harmonic interference strictly synchronized with the workpiece rotation angle; which constitute the core deterministic component of dynamic process error. This paper models this periodic harmonic disturbance as a superposition of multi-order rotational harmonic components:

(8) $$d_{harmonic}(\theta )=d_{0}+\sum_{m=1}^{n}[A_{m}\cos(m\theta -\varphi_{m})]+\varepsilon (\theta )$$

where $d_{harmonic}(\theta )$ denotes the raw measurement signal after structural error compensation; $d_{0}$ denotes the real inner-wall topographical profile; $A_{m}$ and $\varphi_{m}$ denote the amplitude and initial phase of the mth rotational harmonic component, respectively; and ε(θ) denotes the high-frequency random noise component.

Owing to the unknown phase parameter $\varphi_{m}$, this model represents a nonlinear least-squares (NLS) optimization problem; iterative algorithms, when directly applied, are susceptible to issues such as sensitivity to initial values, local optimal minima, and poor convergence. To address this challenge, this paper adopts the Least Squares Harmonic Fitting (LSHF) algorithm [24]. Using trigonometric identities, we expand the nonlinear harmonic terms into a linear combination of orthogonal sine and cosine basis functions. We introduce intermediate parameters $a_{m}=A_{m}\cos\varphi_{m}$ and $b_{m}=A_{m}\sin\varphi_{m}$, which converts the original nonlinear optimization problem into a standard linear least-squares problem:

(9) $$d_{harmonic}(\theta )=d_{0}+\sum_{m=1}^{n}[a_{m}\cos(m\theta )+b_{m}\sin\left(m\theta \right)]+\varepsilon (\theta )$$

Based on the observed data, the algorithm first adaptively determines the optimal harmonic truncation order according to the signal’s spectral characteristics, and subsequently constructs a linear least-squares system. By solving the corresponding normal equations, we directly obtain the globally optimal solution for the linear intermediate parameters to be identified without iterative computation, which fundamentally eliminates the inherent numerical stability issues of nonlinear optimization. Finally, we remove the identified harmonic components from the structural-compensated measurement signal, realizing accurate separation and removal of the deterministic periodic error components:

(10) $$d_{LSHF}(\theta )=d_{harmonic}(\theta )-\sum_{m=1}^{n}[{\hat{a}}_{m}\cos(m\theta )+{\hat{b}}_{m}\sin\left(m\theta \right)]\approx d_{0}+\varepsilon (\theta )$$

#### 3.3.3. Detail-Preserving Suppression of Random Noise Based on Adaptive Wavelet Thresholding

To further suppress high-frequency random noise remaining after harmonic separation while maximally preserving the real topographical features of the deep-hole inner wall, this paper adopts a wavelet thresholding method with excellent time-frequency localization characteristics. We select the db4 wavelet basis function, which features high regularity and compact support, to perform an N-level discrete wavelet transform (DWT) decomposition on the signal $d_{LSHF}(\theta )$. This process decomposes the signal into low-frequency approximation coefficients representing the real topographical profile of the deep-hole inner wall and high-frequency detail coefficients dominated by the residual high-frequency random noise:

(11) $$d_{LSHF}(\theta )=\sum_{k}c_{N,k}\phi_{N,k}(\theta )+\sum_{n=1}^{N}\sum_{k}d_{n,k}\psi_{n,k}(\theta )$$

where $\phi_{N,k}(\theta )$ and $\psi_{n,k}(\theta )$ denote the scaling function and mother wavelet function of the selected db4 wavelet basis, respectively; $c_{N,k}$ denotes the approximation coefficients at the Nth maximum decomposition level; and $d_{n,k}$ denotes the wavelet detail coefficient at the nth decomposition level (1≤n≤N).

To enhance the adaptability to non-stationary measurement signals and denoising robustness, this paper employs Stein’s Unbiased Risk Estimate (SURE) principle to determine the decomposition level-dependent optimal thresholds $T_{j}$. Combined with the robust Median Absolute Deviation (MAD) method for estimating the noise standard deviation of the high-frequency detail coefficients at each decomposition level, we apply the classic soft thresholding function proposed by Donoho to shrink the high-frequency detail coefficients, yielding a set of denoised coefficients ${\hat{d}}_{n,k}$. Finally, we reconstruct the final high-fidelity inner-wall topography measurement signal via the inverse wavelet transform (IDWT):

(12) $$d_{pure}(\theta )=\sum_{k}c_{N,k}\phi_{N,k}(\theta )+\sum_{n=1}^{N}\sum_{k}{\hat{d}}_{n,k}\psi_{n,k}(\theta )$$

After the wavelet thresholding denoising processing, we effectively suppress the high-frequency broadband random noise in the measurement signal while maximally preserving the topographical details of the deep-hole inner wall. This denoising result provides a high-signal-to-noise ratio (SNR) high-quality data foundation for subsequent high-precision inner-wall topographical reconstruction.

## 4. Measurement Experiments and Result Analysis

### 4.1. Experimental Platform Construction

**Figure 6:**
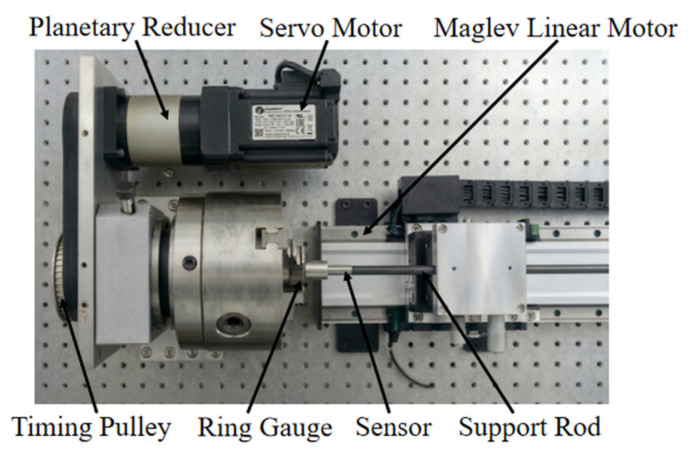
Photograph of the high-precision experimental platform for spectral confocal deep hole inner wall measurement.

To verify the effectiveness of the proposed calibration method and the progressive error compensation system, we developed a dedicated high-precision experimental platform for deep-hole inner-wall topography measurement based on the spectral confocal principle described in Section 2.1, as shown in Figure 6. The platform integrates three core functional units: a high-precision rotary-feed motion module, a high-precision spectral confocal sensing module, and a high-stability support module. The key specifications and design principles of the core components of the platform are detailed as follows:

The axial feed unit employs a maglev linear motor equipped with a high-resolution linear encoder, offering a motion resolution of better than 0.1 μm, a positioning accuracy of ±2 μm, and a positioning repeatability of 1 μm, thereby providing a precise reference for axial positioning over the entire hole depth. The core topography measurement unit employs a CRL1500N spectral confocal displacement sensor (Lingguang Automation Technology Co., Ltd., Yueqing, Zhejiang Province, China), which achieves a linearity of ±0.75 μm within a measurement range of 2.25 mm to 3.75 mm and a measurement repeatability of 0.1 μm, providing high-precision raw data for radial dimension inspection of the deep-hole inner wall. A T700-grade carbon fiber (Jiangxi Shanmeibang Fiber Products Co., Ltd., Ji’an, China) tube with high specific stiffness and a low coefficient of thermal expansion is employed as the hollow support rod for the spectral confocal sensor, effectively suppressing measurement interference caused by mechanical vibrations and temperature fluctuations. The rotary drive unit comprises a two-stage transmission chain composed of a high-precision servo motor, a planetary gear reducer, and a synchronous belt pulley; this transmission configuration ensures that the workpiece rotates continuously at a constant low speed with smooth, impact-free motion, thereby minimizing the rotational harmonic interference introduced during rotation. Furthermore, to minimize the impact of external environmental disturbances on the high-precision measurements, all experiments were conducted in a temperature-controlled metrology laboratory (20 ± 0.5 °C), with the entire measurement system mounted on a vibration-isolation platform.

### 4.2. Geometric Parameter Calibration and Verification Based on Step Ring Gauge

#### 4.2.1. Calibration Experiment and Fitting Process

To determine the intrinsic geometric parameters of the measurement system (radial eccentricity r and combined angle β) and verify the robustness and identification accuracy of the global calibration method proposed in this paper, we performed calibration experiments using a stepped ring gauge. For the experiments we adopted a coaxial mounting configuration identical to that adopted in actual deep-hole measurement scenarios, in which a “standard ring gauge + stepped ring gauge” combined assembly was coaxially mounted on the measurement spindle. The stepped ring gauge serves as the calibration standard and features three concentric inner cylindrical surfaces with nominal diameters of 6.9990 mm, 8.0010 mm, and 9.0010 mm (expanded uncertainty U = 1.2 μm, k = 2); a standard ring gauge with a nominal diameter of 7.9990 mm is used as an independent verification reference to verify that the identified parameters can accurately characterize the relative pose deviation between the measurement axis of the sensor and the theoretical axis of the workpiece.

**Figure 7:**
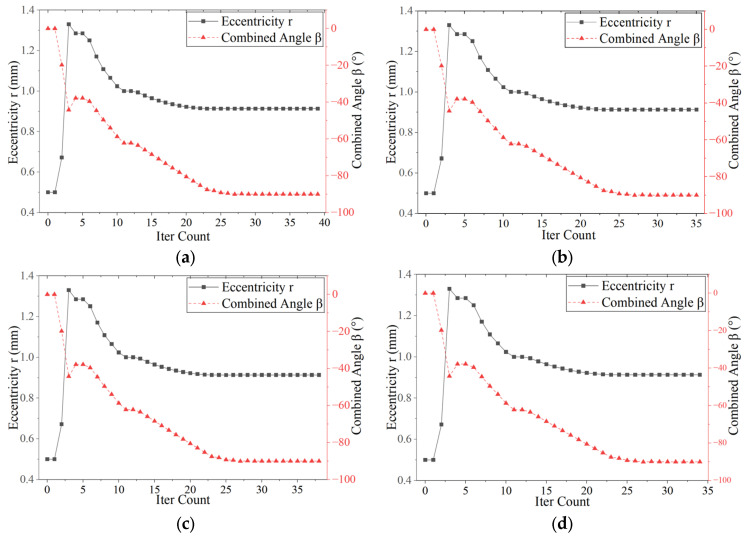
Iterative convergence curves of geometric parameters solved by Levenberg–Marquardt algorithm under different measurement orientations: (**a**) 0° measurement orientation; (**b**) 90° measurement orientation; (**c**) 180° measurement orientation; (**d**) 270° measurement orientation.

To mitigate random measurement uncertainty caused by single-azimuth sampling, we collected calibration data at four discrete azimuths: 0°, 90°, 180°, and 270°. At each azimuth, we programmed the sensor to scan along the same axial generatrix of each stepped inner cylindrical surface, yielding multiple sets of measurement distance versus reference radius data pairs ($d_{ij},{\;R}_{i}$). After preprocessing the data via wavelet thresholding and based on the geometric deviation model established in Section 3.1, we applied the LM nonlinear least-squares algorithm to independently solve for the intrinsic geometric parameters from the four independent datasets, resulting in four sets of identified parameter values. Figure 7 shows the convergence curves of the LM algorithm for the calibration experiments at the four azimuths. The results demonstrate that the objective function values decreased rapidly and stabilized within a finite number of iterations. In particular, the convergence curves across the four independent spatial orientations exhibit exceptional consistency (appearing almost identical), which directly and visually proves the strong orientation-independence and global robustness of the proposed calibration algorithm. This verifies the excellent numerical stability and high reliability of the proposed parameter identification method.

#### 4.2.2. Calibration Result Verification

After the calibration experiments, we kept fixed the mounting pose of the sensor and the clamping status of the standard ring gauge, and only removed the stepped ring gauge. We then collected inner-wall profile data of the standard ring gauge at the same four measurement azimuths used in the calibration experiments, forming four independent validation samples. We substituted the optimal intrinsic geometric parameters identified at each azimuth into Equation (1) to compensate for the geometric deviation. We then calculated the average compensated inner diameter of the standard ring gauge and compared it with its nominal value to validate the measurement accuracy of the proposed geometric deviation compensation method; the results are summarized in Table 1.

#### 4.2.3. Calibration Result Analysis

The calibration and validation experimental results summarized in Table 1 lead to two core conclusions:

The parameter identification results exhibit excellent repeatability and robustness. The geometric parameters of the system exhibit excellent consistency across the four independent measurement azimuths. Specifically, the identified radial eccentricity values are concentrated within the range of 0.9132–0.9134 mm, with an ultra-low standard deviation of approximately 0.0001 mm, while the combined angle values are concentrated between −90.0000° and −89.9962°, with a standard deviation of only 0.0021°, which reflects minimal dispersion of the identified parameters. These results confirm that the geometric deviation of the system constitutes a stable, inherent systematic error of the system. Furthermore, the proposed global calibration method based on a stepped ring gauge successfully eliminates the inherent limitation of parameter non-uniqueness in traditional single-ring gauge calibration methods, which ensures the identified parameters are directly applicable to geometric deviation compensation over the entire hole depth.

The proposed geometric deviation compensation method achieves sub-micrometer measurement accuracy. After we compensate the measurement data using the calibrated parameters, the maximum absolute deviation between the measured inner diameter of the standard ring gauge and its nominal value is only 0.2 μm, with an average absolute deviation of 0.15 μm, both of which are well within the 5 μm MPE limit specified in ISO 10360-2:2009 [25]. These results comprehensively validate the correctness of the established geometric deviation model and the high identification precision of the proposed global calibration method, which enables robust and accurate correction of the inherent geometric deviations of the system over the full measurement range of the hole depth.

#### 4.2.4. Verification of the Sufficiency of Calibration Angles

Theoretically, the four orthogonal orientations described above form a complete orthogonal basis for the two-dimensional cross-section. This setup fundamentally guarantees the full extraction of all linearly independent components and extremal features of the spatial geometric error vectors. To experimentally verify the sufficiency of this four-angle calibration strategy, supplementary measurements at intermediate angles were conducted. Calibration datasets comprising 4, 8, and 12 angles were then constructed for comparative analysis, with the results summarized in Table 2.

The results demonstrate that, following the introduction of non-orthogonal measurement phases, the maximum variations in the core calibration parameters r and β are merely 0.2 μm and 0.0026°, respectively, both falling strictly within the sub-micrometer range. This experimental evidence strongly confirms that the four-orientation orthogonal sampling strategy is sufficiently precise to capture all extremal spatial features of the physical system. By completely eliminating redundant data coupling and avoiding redundant testing, this strategy successfully achieves an optimal balance between high calibration accuracy and industrial-grade inspection efficiency.

### 4.3. Deep-Hole Workpiece Measurement and Verification of Comprehensive Compensation Effect

To verify the effectiveness of the proposed multi-source error compensation strategy proposed in this paper, we selected a typical industrial deep-hole workpiece with a high L/D ratio for full-depth measurement validation after the system calibration. This experiment adopted the inner diameter deviation and roundness error as the primary evaluation metrics. Through repeated measurements across multiple cross-sections, we systematically evaluated the comprehensive performance of the strategy in mitigating the three core errors—geometric eccentricity error, structural deflection error, and dynamic disturbances error. The test specimen was a 45CrMo alloy deep-hole workpiece with an inner diameter of 8 mm, an outer diameter of 18 mm, and a hole depth of 250 mm. Its L/D ratio exceeds 30, representing a typical small-diameter, high-aspect-ratio structure that poses a significant metrological challenge in practical measurement. We utilized a hollow support rod in the measurement system with a cantilevered length of 350 mm and a sensor with a mass of 23 g mounted at its free end. These experimental conditions closely match the actual industrial measurement scenarios, ensuring the practical significance and engineering applicability of the research conclusions.

#### 4.3.1. Single-Section Comprehensive Compensation Process

Taking the cross-section at an axial depth of 175 mm as an illustrative example, we describe an integrated compensation procedure that is fully consistent with the previously proposed “geometric calibration–structural correction–dynamic filtering” framework. This integrated compensation procedure is applicable to all arbitrary cross-sections over the entire hole depth, and its core steps are detailed as follows:

- Global Calibration of Geometric Parameters

We coaxially mounted the stepped ring gauge on the end face of the measurement workpiece. We controlled the sensor to axially scan the inner-wall profile generatrix of the gauge with a feed rate of 0.5 mm/s and a sampling frequency of 20 Hz. Based on the geometric deviation model in Section 3.1 and the calibration procedure in Section 4.2, we determined the geometric deviation parameters of the system under this specific clamping status as follows: radial eccentricity r = 1.1904 mm and combined angle β = −66.8865°. We then removed the stepped ring gauge after the calibration was completed.

- Structural Error Correction

**Figure 8:**
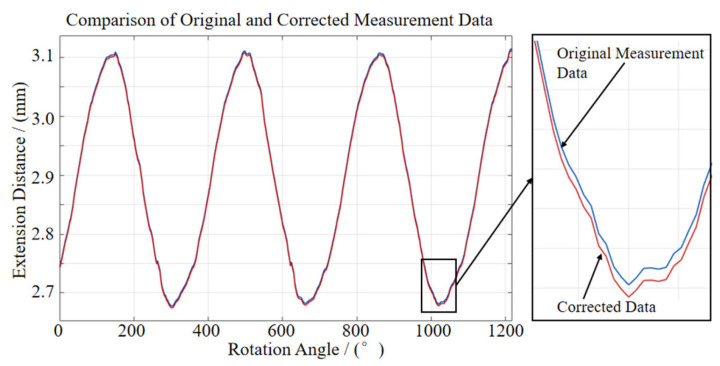
Comparison of radial measurement data before and after structural error correction at 175 mm depth cross-section.

We positioned the sensor at the target cross-section and rotated the workpiece at a constant speed of 10 rpm to acquire the raw contour point-cloud data $d_{raw}(\theta )$ over at least one complete revolution. Subsequently, the raw data were corrected using the static deflection model described in Section 3.2 to eliminate the cumulative systematic errors introduced by the elastic deformations of the support rod and the workpiece. This process yielded the corrected point cloud $d_{struct}(\theta )$ as illustrated in Figure 8.

- Harmonic Error Separation and Noise Suppression

**Figure 9:**
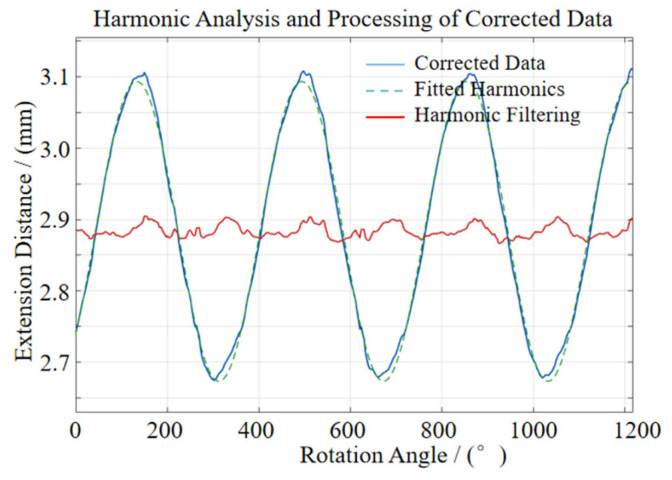
Comparison of measurement signals before and after periodic harmonic error elimination by least square harmonic fitting.

**Figure 10:**
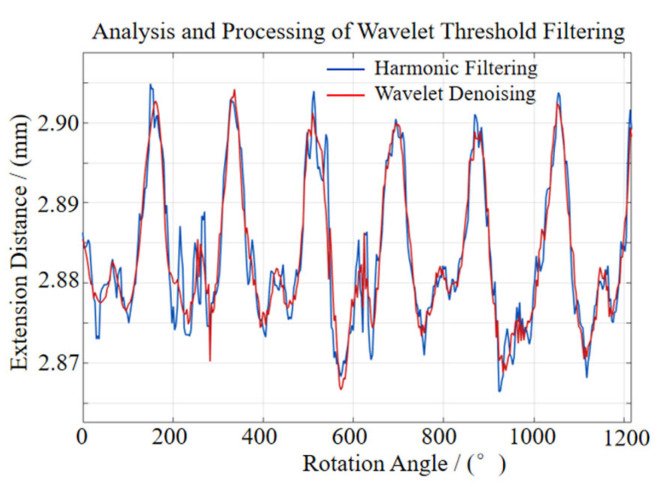
Comparison of measurement signals before and after random noise suppression by adaptive wavelet threshold filtering.

We utilized the least-squares harmonic fitting (LSHF) method to identify and subtract the deterministic periodic harmonic interference components introduced by the rotary drive train from $d_{struct}(\theta )$, yielding the purified point cloud $d_{LSHF}(\theta )$ (Figure 9). Subsequently, we applied adaptive wavelet thresholding to suppress residual high-frequency random measurement noise, resulting in the final high-fidelity contour point cloud $d_{pure}(\theta )$, as illustrated in Figure 10.

- Morphology Parameter Evaluation

**Figure 11:**
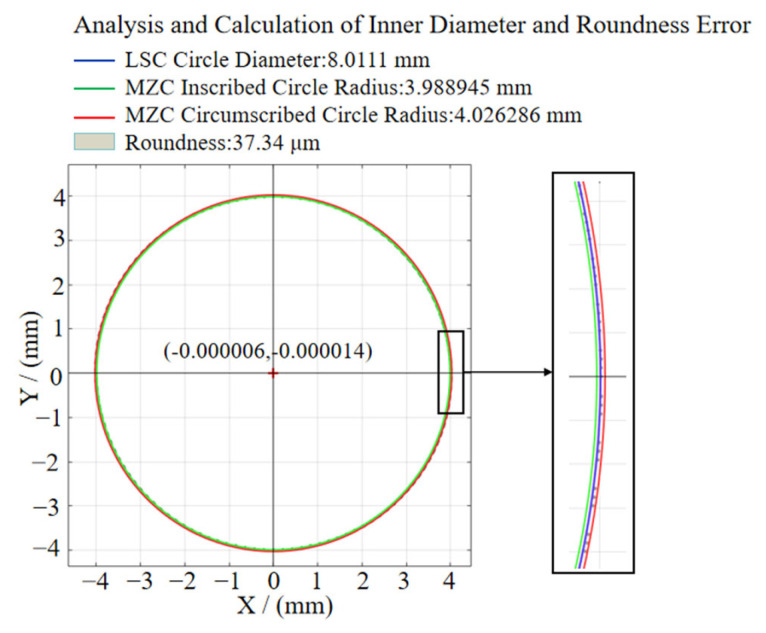
Fitting result of least square circle and roundness evaluation result by minimum zone circle method at 175 mm depth cross-section after full-process compensation.

By incorporating the calibrated geometric parameters, we substituted $d_{pure}(\theta )$ into Equation (1) to reconstruct the true radius of the measured cross-section. The inner diameter was calculated to be 8.0111 mm via the least-squares circle (LSC) method [26], while the roundness error was evaluated as 37.34 μm using the minimum zone circle (MZC) method [27], as illustrated in Figure 11.

#### 4.3.2. Verification of Multi-Section Collaborative Compensation for Deep Holes

To quantitatively evaluate the adaptability of the compensation strategy over the entire hole depth and the measurement repeatability, we conducted experiments at four characteristic cross-sections (Z = 75 mm, 125 mm, 175 mm and 225 mm) along the workpiece axis. We performed all measurements sequentially under a single clamping configuration, strictly adhering to the calibration and compensation procedures outlined in Section 4.3.1. To assess repeatability, we independently repeated the integrated “clamping–calibration–measurement–compensation” workflow ten times. The statistical results for inner diameter and roundness error measurements after full-process compensation are summarized in Table 3 and Table 4, respectively. Furthermore, in order to quantitatively reveal the dynamic evolution of multi-source errors along the hole depth, Table 5 presents an assessment of the quantitative contributions of geometric deviation, structural error, and dynamic process error at each cross-section.

#### 4.3.3. Measurement Result Analysis

The data obtained from repeated measurements at multiple cross-sections yielded two principal conclusions:

Inner diameter measurements confirm that the proposed method achieves superior accuracy and repeatability over the entire hole depth. After comprehensive compensation, the deviations between the mean inner diameter measurements at the four axial cross-sections and the nominal diameter of 8.0 mm (IT7 grade, 15 μm tolerance) were in the range of 10.4 to 16.6 μm. This aligns with actual workpiece machining tolerances, demonstrating that multi-source errors have been effectively suppressed and precisely compensated. Furthermore, the standard deviations of repeated measurements at each cross-section remained stable between 0.8 and 1.6 μm, showing no observable degradation as a function of measurement depth. These results validate the global applicability of the proposed calibration method and the effectiveness of the structural deflection correction model, thereby addressing the core engineering challenge of measurement accuracy degradation in deep holes with high L/D ratios.

Roundness error measurements validate the high-fidelity topography reconstruction and measurement robustness of the proposed method. Following compensation, the mean roundness error across the four cross-sections exhibited a gradual decreasing trend with increasing axial depth. This aligns closely with the inherent characteristics of the deep-hole drilling process, where tool vibration diminishes as the hole deepens, resulting in a roundness error at the bottom that is lower than at the entrance. These results demonstrate that the compensated measurement signals have effectively eliminated systematic errors, accurately reproducing the true topographical features of the workpiece. The standard deviations of the roundness error from repeated measurements remained stable between 2.8 and 5.41 μm, confirming excellent measurement stability over the entire hole depth. This fully validates the effectiveness of the dynamic error suppression strategy and provides a reliable technical basis for the quantitative assessment of geometric tolerances in deep-hole machining.

The measurement uncertainty for the inner diameter was evaluated in accordance with ISO Guide 98-3:2008 (Evaluation and Expression of Measurement Uncertainty) [28]. The primary sources of uncertainty identified include repeated measurements, stepped ring gauge calibration, sensor measurement error, residual errors from geometric parameter calibration, residual errors from structural and dynamic compensation (with a maximum residual amplitude bounded within 0.5 μm), and ambient temperature fluctuations. Following a combined evaluation, the expanded uncertainty for the inner diameter measurement is U = 3.8 μm, k = 2. This indicates that the measurement results obtained in this study exhibit high metrological credibility, satisfying the industrial precision inspection requirements for deep holes with a high L/D ratio at IT7 grade accuracy.

## 5. Conclusions

This study proposes a systematic calibration and error compensation framework to address the issues of uneven measurement accuracy and topographical distortion in spectral confocal deep-hole measurement induced by multi-source errors. By analyzing the underlying mechanisms and propagation patterns, we categorize the core measurement errors into three types—geometric deviation, structural error, and dynamic process error—corresponding to the propagation sequence of “fixed system error–cumulative depth-dependent error–dynamic disturbance.” We establish a progressive compensation system that integrates geometric calibration, structural correction, and dynamic filtering. The three primary technological innovations presented are: (1) a global calibration method based on stepped ring gauge and Levenberg–Marquardt (LM) optimization algorithms, which overcomes the intrinsic parameter non-uniqueness limitation of traditional single-ring gauge calibration to achieve sub-micrometer-level correction accuracy of geometric deviations; (2) a comprehensive static deflection model for the support rod and workpiece, enabling quantitative correction of gravity-induced structural deflection errors and suppressing accuracy degradation as hole depth increases; (3) a combined approach of least-squares harmonic fitting and adaptive wavelet thresholding to isolate periodic harmonic errors and suppress random noise, realizing high-fidelity inner-wall topography reconstruction. Experimental results demonstrate that the maximum absolute deviation for standard ring gauge diameter measurement is 0.2 μm, significantly outperforming the 5 μm maximum permissible error (MPE) limit specified in ISO 10360-2:2009. For deep-hole workpieces with L/D ratios exceeding 30, the inner diameter measurement repeatability remains stable at 0.8–1.6 μm, with an expanded uncertainty of U = 3.8 μm (k = 2) and no observable depth-dependent decay. The roundness error measurements align closely with inherent deep-hole machining characteristics, accurately reflecting the geometric features of the inner wall.

This method significantly improves measurement accuracy through algorithmic optimization without altering the hardware, providing a low-cost and highly versatile solution. Although it has been validated on workpieces with a diameter of 8 mm and an L/D ratio of 30:1, further experiments involving diverse hole diameters and materials are required in future research to fully verify its universality.

## Figures and Tables

**Table 1:** Calibration results of system geometric parameters and verification accuracy under different measurement orientations.

Orientation (°)	Eccentricity (mm)	Combined Angle (°)	Compensated Inner Diameter (mm)	Reference Inner Diameter (mm)	Absolute Deviation (μm)
0	0.9132	−90.0000	7.9988	7.9990	0.2
90	0.9134	−89.9962	7.9991	7.9990	0.1
180	0.9134	−89.9996	7.9992	7.9990	0.2
270	0.9133	−89.9962	7.9989	7.9990	0.1
Mean	0.9133	−89.9980	7.9990	-	0.15
Std. Dev.	0.0001	0.0021	0.0002	-	0.06

**Table 2:** Experimental comparison of calibration results with different angle configurations.

Number of Orientations	Eccentricity (mm)	Combined Angle (°)	Compensated Inner Diameter (mm)	Reference Inner Diameter (mm)	Absolute Deviation (μm)
4	1.0241	−74.9989	7.9988	7.9990	0.2
8	1.0243	−74.9982	7.9992	7.9990	0.2
12	1.0242	−74.9995	7.9990	7.9990	0
Max. Variation	0.0002	0.0013	0.0004	-	0.2

**Table 3:** Statistical results of measured inner diameter for each axial cross-section after full-process compensation (unit: mm).

Test No.	Inner Diameter (75 mm)	Inner Diameter (125 mm)	Inner Diameter (175 mm)	Inner Diameter (225 mm)
1	8.0115	8.0161	8.0111	8.0123
2	8.0095	8.0187	8.0145	8.0122
3	8.0104	8.0162	8.0157	8.0101
4	8.0100	8.0168	8.0134	8.0114
5	8.0108	8.0134	8.0149	8.0126
6	8.0088	8.0177	8.0146	8.0141
7	8.0110	8.0174	8.0135	8.0134
8	8.0099	8.0169	8.0121	8.0113
9	8.0109	8.0170	8.0133	8.0132
10	8.0111	8.0157	8.0110	8.0137
Mean	8.0104	8.0166	8.0134	8.0124
Std. Dev.	0.0008	0.0014	0.0016	0.0012

**Table 4:** Statistical results of measured roundness error for each axial cross-section after full-process compensation (unit: μm).

Test No.	Roundness Error (75 mm)	Roundness Error (125 mm)	Roundness Error (175 mm)	Roundness Error (225 mm)
1	42.42	39.70	37.34	36.66
2	43.43	40.42	35.57	44.09
3	39.57	39.40	35.70	33.17
4	41.45	36.74	37.83	35.00
5	24.98	43.04	33.70	37.24
6	37.76	36.27	35.21	27.07
7	40.79	34.07	36.83	34.77
8	39.97	35.14	45.07	31.31
9	37.18	40.57	33.29	45.15
10	39.56	37.29	40.80	36.04
Mean	38.71	38.26	37.14	36.05
Std. Dev.	5.19	2.80	3.52	5.41

**Table 5:** Quantitative evaluation of multi-source error contributions at different axial cross-sections (unit: μm).

Axial Depth *Z* (mm)	Geometric Deviation	Structural Error	Dynamic Error	Total Error
75	1122.32 (90.40%)	0.55 (0.04%)	118.67 (9.56%)	1241.54 (100%)
125	1122.33 (86.63%)	1.67 (0.13%)	171.48 (13.24%)	1295.48 (100%)
175	1122.31 (83.83%)	3.09 (0.23%)	213.33 (15.94%)	1338.73 (100%)
225	1122.33 (78.05%)	4.63 (0.33%)	310.94 (21.62%)	1437.90 (100%)

## Data Availability

The original contributions presented in this study are included in the article. Further inquiries can be directed to the corresponding author.
